# Late presentation of chronic viral hepatitis for medical care: a consensus definition

**DOI:** 10.1186/s12916-017-0856-y

**Published:** 2017-05-03

**Authors:** Stefan Mauss, Stanislas Pol, Maria Buti, Erika Duffell, Charles Gore, Jeffrey V. Lazarus, Hilje Logtenberg-van der Grient, Jens Lundgren, Antons Mozalevskis, Dorthe Raben, Eberhard Schatz, Stefan Wiktor, Jürgen K. Rockstroh, on behalf of the European consensus working group on late presentation for Viral Hepatitis Care, Stanislas Pol, Maria Buti, Stefan Mauss, Erika Duffell, Stefan Wiktor, Antons Mozalevskis, Irene Veldhuijzen, Nikos Dedes, Charles Gore, Eberhard Schatz, José Gatell, Jeffrey V. Lazarus, Jens Lundgren, Dorthe Raben, Jürgen Rockstroh, Brian West, Jordi Casabona, Nikos Dedes

**Affiliations:** 1 Center for HIV and Hepatogastroenterology, Humboldtstrasse 18, 40237 Düsseldorf, Germany; 2 European Association for the Study of the Liver (EASL), 7rue Daubin, 1203 Geneva, Switzerland; 3 Liver Unit Hospital Universitari Vall d’Hebron and Centro de Investigación Biomédica en Red (CIBER) de Enfermedades Hepáticas y Digestivas (CIBERehd)del Instituto Carlos III Barcelona, Paseo Valle Hebron 127, Barcelona, 08035 Spain; 4 European Centre for Disease Prevention and Control (ECDC), Granits väg 8, 171 65 Solna, Sweden; 5 Hepatitis C Trust, World Hepatitis Alliance, 27 Crosby Row, London, SE1 3YD UK; 6 CHIP, Department of Infectious Diseases Section 2100, Rigshospitalet, University of Copenhagen, Finsencentret, Blegdamsvej 9, DK-2100 Copenhagen, Denmark; 7 European Liver Patients Association (ELPA), Rue de la Loi 235/27, 1040 Brussels, Belgium; 8 World Health Organization (WHO) Regional Office for Europe, UN City Marmorvej 51, DK-2100 Copenhagen, Denmark; 9 Liver Department, Cochin Hospital, 27, rue du Faubourg-Saint-Jacques, 75014 Paris, France; 10 HIV in Europe, Section 2100, Finsencentret Blegdamsvej 9, DK-2100 Copenhagen, Denmark; 11 Foundation De Regenboog Groep (FRG) representing Correlation Network, Hepatitis C Initiative, Postbus 10887, 1001 EW Amsterdam, The Netherlands; 12 Global Hepatitis Programme, World Health Organization (WHO), Geneva, Switzerland; 13 Department of Medicine I, University of Bonn, Sigmund-Freud-Str. 25, D-53105 Bonn, Germany

**Keywords:** Late presentation, Viral hepatitis, Testing

## Abstract

**Introduction:**

We present two consensus definitions of advanced and late stage liver disease being used as epidemiological tools. These definitions can be applied to assess the morbidity caused by liver diseases in different health care systems. We focus is on hepatitis B and C virus infections, because effective and well tolerated treatments for both of these infections have greatly improved our ability to successfully treat and prevent advanced and late stage disease, especially if diagnosed early. A consensus definition of late presentation with viral hepatitis is important to create a homogenous, easy-to-use reference for public health authorities in Europe and elsewhere to better assess the clinical situation on a population basis.

**Methods:**

A working group including viral hepatitis experts from the European Association for the Study of the Liver, experts from the HIV in Europe Initiative, and relevant stakeholders including patient advocacy groups, health policy-makers, international health organisations and surveillance experts, met in 2014 and 2015 to develop a draft consensus definition of late presentation with viral hepatitis for medical care. This was refined through subsequent consultations among the group.

**Results:**

Two definitions were agreed upon. Presentation with advanced liver disease caused by chronic viral hepatitis for medical care is defined as a patient with chronic hepatitis B and C and significant fibrosis (≥ F3 assessed by either APRI score > 1.5, FIB-4 > 3.25, Fibrotest > 0.59 or alternatively transient elastography (FibroScan) > 9.5 kPa or liver biopsy ≥ METAVIR stage F3) with no previous antiviral treatment. Late stage liver disease caused by chronic viral hepatitis is clinically defined by the presence of decompensated cirrhosis (at least one symptom of the following: jaundice, hepatic encephalopathy, clinically detectable ascites, variceal bleeding) and/or hepatocellular carcinoma.

**Conclusion:**

These consensus definitions will help to improve epidemiological understanding of viral hepatitis and possibly other liver diseases, as well as testing policies and strategies.

## Introduction

Over 13 million adults are living with hepatitis B, and 15 million with hepatitis C, in the World Health Organization (WHO) European Region [1–4]. The prevalence of chronic hepatitis B virus (HBV) infection (commonly defined as the persistence of hepatitis B surface antigen for six months or more) and chronic hepatitis C virus (HCV) infection (as determined by the persistence of hepatitis C nucleic acid or HCV core antigen for more than six or 12^1^ months) ranges from 0.1% to 6% across Europe, with major differences between countries and population subgroups [2–4]. Chronic HBV and HCV infections may remain clinically silent for decades, and symptoms do occur at a late stage. Diagnosis in the absence of widespread screening programmes may therefore be based on signs of late stage liver disease such as hepatic decompensation, variceal bleeding or hepatocellular carcinoma.

Many people with chronic HBV and/or HCV infection are go undiagnosed [5]. Of those already diagnosed, many are not necessarily linked to parts of the healthcare system that are able to provide comprehensive care (e.g. to accurately classify the extent of liver disease and provide treatment when indicated) [6]. Consequently, a large (but undetermined) proportion of the chronically infected population enters comprehensive care only after developing liver disease-related clinical symptoms.

Effective and well tolerated treatments for both HBV and HCV infection have greatly improved our ability treat patients successfully, especially if they are diagnosed early [7–10]. In asymptomatic individuals, treatment is indicated for those at increased risk of symptomatic chronic liver disease, and those at risk of transmitting the infection. All patients with symptomatic disease should be treated. For many, treatment can prevent further progression of liver disease to liver cirrhosis, and can even revert existing liver fibrosis [7, 8].

In most European countries, it remains unknown as to what extent testing policies and strategies succeed in identifying the undiagnosed population during the course of their disease. The extent to which diagnosed patients are linked to and retained in sections of the healthcare system that are able to provide comprehensive care is also unknown.

To fully exploit the strategic use of treatment and to optimise its benefit, infected persons in need of treatment must enter comprehensive care before their liver disease progresses to considerable liver damage. Patients with advanced liver fibrosis may be considered as “late presenters”. Of these, a subgroup of individuals with “late stage liver disease”, such as decompensated liver cirrhosis, portal hypertension or hepatocellular carcinoma, can be further defined as a subgroup where there is indisputable evidence that earlier initiation of treatment would have provided significant benefit. These definitions will help quantify the proportion of cases missing timely diagnosis and treatment.

## Method/process of developing a consensus definition of late presentation with viral hepatitis

In 2014, a group of viral hepatitis experts within the European Association for the Study of the Liver (EASL) and the HIV in Europe Initiative [11] formed a working group to develop a consensus definition of late presentation with viral hepatitis. Key stakeholders were invited to participate, including patient advocacy groups, health policy-makers, international health organisations, surveillance experts and medical experts. The consensus-building process involved all of the important constituencies in Europe involved in both treatment and surveillance of hepatitis. A series of teleconferences took place in 2014, in parallel with the organisation of the first HepHIV Conference in Barcelona in October 2014, where the first draft of the definition for late presentation was presented and discussed [12]. Following the conference, key stakeholders were consulted on the proposed consensus definitions in a public hearing phase. The definitions were finally endorsed by the EASL governing board in September 2015.

## Results

Two definitions relating to late presentation were agreed upon (Table 1).

**Table 1:** Consensus definition of late presentation with chronic viral hepatitis for medical care

Definition	Description
Presentation with advanced liver disease in untreated patients with chronic hepatitis B and C	A patient with chronic hepatitis B or C and significant fibrosis assessed by one of the following: serologic fibrosis score ≥ F3 (assessed by APRI score > 1.5, FIB-4 > 3.25, Fibrotest > 0.59 or alternatively a transient elastography (FibroScan) > 9.5 kPa) or liver biopsy (≥ METAVIR stage F3) in patients with no previous antiviral treatment^a^.
Presentation with late stage liver disease in untreated patients with chronic hepatitis B and C	Presence of at least one symptom of decompensated cirrhosis (jaundice, hepatic encephalopathy, clinically detectable ascites, variceal bleeding) and/or hepatocellular carcinoma in patients with no previous antiviral treatment^b^.

^a^General comments: advanced liver disease in patients with chronic hepatitis B and C is a definition for capturing all cirrhotics and patients with pre-cirrhosis. On the basis of regular testing of the aspartate transaminase level (AST), alanine transaminase level (ALT), gamma glutamyl transferase level (GGT), cholesterol and platelet count, it is possible to calculate the aspartate transaminase to platelets radio index (APRI) as: APRI = (AST/upper limit of normal [ULN])/platelet (109/L) x 100), or to calculate the FIB-4 as FIB-4 = (age x AST) / (platelets x (sqr (ALT) or with more extensive laboratory assessments the commercially available Fibrotest. APRI and FIB-4 may have lower sensitivity and specifity in calculating advanced fibrosis and cirrhosis patients with chronic hepatitis B compared to chronic hepatitis C. The APRI score may not be reliable if a patient suffers from another condition that affects the platelet count (e.g. HIV infection, immune thrombopenia etc), and therefore transient elastography (e.g. FibroScan) is preferred.

^b^In the absence of other explanatory factors/aetiologies aside HBV, HCV or HDV

The term “late presentation for care” should be used to refer to HBV or HCV-infected people who enter care when substantial liver fibrosis is already present (i.e. they present with advanced liver disease). This implies that the time of HBV or HCV diagnosis is considered late, as “late presenters” have not been diagnosed earlier. In contrast, the term “presentation with late stage liver disease” should be reserved for the subgroup of these late presenters who are additionally at greater imminent risk of severe complications of liver disease or death. The term “presentation for care” means attendance at a healthcare facility that is able to monitor progression of chronic hepatitis B and C and associated liver disease and initiate appropriate medical care, including treatment.

## Discussion

These consensus definitions may be considered for inclusion within countries’ routine viral hepatitis surveillance systems. Investigations performed on the basis of a common definition will enable epidemiological data to be compared between countries and trends to be monitored over time.

For this purpose the definition of “presentation with advanced liver disease in patients with chronic hepatitis B and C” includes several different technical procedures to estimate the degree of liver fibrosis to improve its practicality, which all have different sensitivities and specificities [13, 14]. In particular, the inclusion of APRI and FIB-4 should enable this definition to be used on a broad scale, and also in low-income countries. However, since the accuracy of APRI in assessing fibrosis in HBV infection has been challenged [15], APRI should only be used in chronic hepatitis B in the absence of other tools including FIB-4. Using a uniform cut-off for the recommended tests for chronic hepatitis B and C may lead to a loss in accuracy [15–17], but is in line with current WHO recommendations [9, 10]. In addition, using the same cut-offs for chronic hepatitis B and C increases the practicality of this definition as a population-based tool.

The second definition of “presentation with late stage liver disease in patients with chronic hepatitis B and C” is based on clinical symptoms alone, with no need for sophisticated technology. This enables its use in any health care system. In some patients, particularly those with chronic hepatitis B, hepatocellular carcinoma may occur without cirrhosis, but usually after prolonged periods of chronic infection [18].

The two key indicators to be derived from using the two definitions of late presentation of patients for medical care with chronic hepatitis B and C within a population of new referrals are: 1) the proportion of referrals that fulfil either of these definitions, and 2) the incidence of presenters with late stage liver disease in the population.

If the definitions are implemented in surveillance structures, the data on which these definitions are based must be readily available from routine care in centres that are specialised to diagnose and treat liver diseases. To achieve this, these centres must adequately capture data on liver fibrosis stage and presence of hepatocellular carcinoma or decompensated cirrhosis.

It is important that viral hepatitis surveillance systems capture the public health consequences of these infections by focusing on the proportion of patients referred to a specialised medical site who present late and/or with advanced liver disease. In the past, this was demonstrated by the introduction of a comparable definition of late presentation with HIV. The broad acceptance of this definition (defined as individuals newly presenting for HIV care with a CD4 count below 350 cells/μl, or with an AIDS-defining event) has allowed the percentages of late presenters in various countries and regions to be compared, and also allows changes in the numbers of late presenters to be monitored after implementing improved testing strategies [19, 20].

Using this definition has been particularly instrumental in identifying risk factors for late presentation, and therefore has had an impact on new testing strategies. Indeed, a recent Swiss cohort analysis showed that patients outside established HIV risk groups are most likely to be late presenters. Provider-initiated testing must therefore be improved to reach these groups, which include heterosexual men and women, and older patients [21].

The late presenter definition has also been used to characterize a specific group of HIV patients with prolonged low CD4-positive cell counts, who behave very differently to other HIV-infected patient groups. More recently, a study on non-infectious comorbidities revealed that these were also far more prevalent in late presenters [22]. In summary, the definition of late presentation has been instrumental in better understanding clinical presentation, course and epidemiology of HIV in various regions of the world.

The two definitions presented here for liver disease in patients with chronic hepatitis B and C can be used for different purposes. Firstly, they will unify methods of monitoring and evaluating the effectiveness of testing and referral services. For example, if a large percentage of patients are “late presenters”, it implies that intervention testing needs improvement to ensure earlier diagnosis. As such, the definitions can be used to monitor the effect of interventions that aim to reduce the number of late presenters. Secondly, their use will enable future studies across Europe to determine the size of the population at risk, and to identify vulnerable groups and risk factors for late presentation. They will also increase understanding of the social and medical barriers that limit access to healthcare in different European countries, and may initiate studies on access to treatment for late presenters across the region. It would therefore be beneficial if all national health agencies, institutions and researchers could implement these consensus definitions when reporting surveillance or research data on late presentation of chronic hepatitis B or C.

These consensus definitions of late presentation for viral hepatitis provide a useful tool for public health authorities in Europe and elsewhere, to gain a better understanding of epidemics. They will help to improve the quality of available epidemiological information on viral hepatitis and the prevention and control responses to the viral hepatitis epidemic.

## Abbreviations

EASL: European Association for the Study of the Liver
HBV: Hepatitis B Virus
HCV: Hepatitis C Virus
HDV: Hepatitis D Virus
WHO: World Health Organization

## Footnotes

1 Case definitions for hepatitis B and C vary across European countries. Countries in the European Union (EU) and European Economic Area (EEA) are requested to follow EU 2012 case definitions for reporting at the European level. 2012/506/EC – Commission Implementing Decision of 8 August 2012 amending Decision 2002/253/EC laying down case definitions for reporting communicable diseases to the Community network under Decision No 2119/98/EC of the European Parliament and of the Council.

## References

[1] World Health Organization Regional Office for Europe. Hepatitis C in the WHO European region. Fact sheet July 2015 [Internet]. World Health Organization; 2015. http://www.euro.who.int/__data/assets/pdf_file/0010/283357/fact-sheet-en-hep-c.pdf?ua=1. Accessed [03 Apr 2017].

[2] Hope VD, Eramova I, Capurro D, Donoghoe MC (2014). Prevalence and estimation of hepatitis B and C infections in the WHO European region: a review of data focusing on the countries outside the European Union and the European Free Trade Association. Epidemiol Infect.

[3] European Centre for Disease Prevention and Control (ECDC). Hepatitis B surveillance in Europe 2013. Stockholm: ECDC; 2015. http://ecdc.europa.eu/en/publications/publications/hepatitis-b-surveillance-in-europe-2013.pdf. Accessed [03 Apr 2017].

[4] European Centre for Disease Prevention and Control. Hepatitis C surveillance in Europe 2013. Stockholm: ECDC; 2015. http://www.euro.who.int/__data/assets/pdf_file/0010/283357/fact-sheet-en-hep-c.pdf?ua=1. Accessed [03 Apr 2017].

[5] Hatzakis A, Wait S, Bruix J, Buti M, Carballo M, Cavaleri M (2011). The state of hepatitis B and C in Europe: report from the Hepatitis B and C Summit Conference. J Viral Hepat.

[6] Blachier M, Leleu H, Peck-Radosavljevic M, Valla DC, Roudot-Thoraval F (2013). The burden of liver disease in Europe: a review of available epidemiological data. J Hepatol.

[7] American Association for the Study of Liver Diseases (AASLD)/Infectious Diseases Society of America (IDSA). When and in whom to initiae HCV therapy. In: HCV guidance: recommendations for testing, managing, and treating hepatitis C. AASLD/IDSA; 2015. http://www.hcvguidelines.org/full-report/when-and-whom-initiate-hcv-therapy. Accessed 4 Jan 2016.

[8] European Association for the Study of the Liver (EASL) (2015). EASL recommendations on treatment of hepatitis C. J Hepatol.

[9] World Health Organization (2015). Guidelines for the prevention, care and treatment of persons with chronic hepatitis B infection.

[10] World Health Organization (2014). Guidelines for the screening, care and treatment of persons with hepatitis C infection.

[11] HIV in Europe Initiative. http://www.hiveurope.eu. Accessed [03 Apr 2017].

[12] Buti M, Pol S, Walker M, on behalf of the working group (2014). A consensus definition of late presentation of viral hepatitis for medical care.

[13] Castera L (2012). Noninvasive methods to assess liver disease in patients with hepatitis B or C. Gastroenterology.

[14] Degos F, Perez P, Roche B, Mahmoudi A, Asselineau J, Voitot H (2010). Diagnostic accuracy of FibroScan and comparison to liver fibrosis biomarkers in chronic viral hepatitis: a multicenter prospective study (the FIBROSTIC study). J Hepatol.

[15] Kim WR, Berg T, Asselah T, Flisiak R, Fung S, Gordon SC (2016). Evaluation of APRI and FIB-4 scoring systems for non-invasive assessment of hepatic fibrosis in chronic hepatitis B patients. J Hepatol.

[16] Teshale E, Lu M, Rupp LB, Holmberg SD, Moorman AC, Spradling P (2014). APRI and FIB-4 are good predictors of the stage of liver fibrosis in chronic hepatitis B: the Chronic Hepatitis Cohort Study (CHeCS). J Viral Hepat.

[17] Zhu MY, Zou X, Li Q, Yu DM, Yang ZT, Huang D, et al. A novel noninvasive algorithm for the assessment of liver fibrosis in patients with chronic hepatitis B virus infection. J Viral Hepat. 2017; doi:10.1111/jvh.12682. 10.1111/jvh.12682 28130852

[18] Chen CF, Lee WC, Yang HI, Chang HC, Jen CL, Iloeje UH (2011). Changes in serum levels of HBV DNA and alanine aminotransferase determine risk for hepatocellular carcinoma. Gastroenterology.

[19] Late presenters working group in COHERE in EuroCoord, Mocroft A, Lundgren J, Antinori A, Monforte Ad, Brännström J, et al. Late presentation for HIV care across Europe: update from the Collaboration of Observational HIV Epidemiological Research Europe (COHERE) study, 2010 to 2013. Euro Surveill. 2015;20(47). http://www.eurosurveillance.org/ViewArticle.aspx?ArticleId=21315. 10.2807/1560-7917.ES.2015.20.47.30070 26624933

[20] Mocroft A, Lundgren JD, Sabin ML, Monforte A, Brockmeyer N, Casabona J (2013). Risk factors and outcomes for late presentation for HIV-positive persons in Europe: results from the Collaboration of Observational HIV Epidemiological Research Europe Study (COHERE). PLoS Med.

[21] Darling KE, Hachfeld A, Cavassini M, Kirk O, Furrer H, Wandeler G (2016). Late presentation to HIV care despite good access to health services: current epidemiological trends and how to do better. Swiss Med Wkly.

[22] Guaraldi G, Zona S, Menozzi M, Brothers TD, Carli F, Stentarelli C (2017). Late presentation increases risk and costs of non-infectious comorbidities in people with HIV: an Italian cost impact study. AIDS Res Ther.

